# Drone-based dataset of annotated sunflower images from Bangladesh

**DOI:** 10.1016/j.dib.2025.111417

**Published:** 2025-02-21

**Authors:** Md. Shafayat Hossain, Mohammad Rifat Ahmmad Rashid, Md. Fahim, Tahzib-E-Alindo, Md Sawkat Ali, Maheen Islam, Mohammad Manzurul Islam, Md. Hasanul Ferdaus, Nishat Tasnim Niloy

**Affiliations:** Department of Computer Science and Engineering, East West University, Aftabnagar, Dhaka, Bangladesh

**Keywords:** Sunflower detection, Object detection, Yield prediction, Semi-supervised learning, Image processing, Computer vision

## Abstract

Accurate and automated detection of sunflower plants, along with assessments of their growth stages and health conditions, is crucial for enabling precision agriculture and improving crop management. In this work, we present a drone-based dataset of annotated sunflower images, derived from high-resolution videos captured at two distinct locations in Bangladesh. The original dataset comprises 1649 images extracted from drone footage of the BARI Surjomukhi-3 variety under various orientations, health conditions, and weather scenarios. After meticulous annotation using the Roboflow platform and augmentation with seven distinct techniques, the dataset expanded to 4286 images in Pascal VOC format. Detailed metadata—including geospatial coordinates, timestamped acquisition conditions, and camera settings—accompanies the dataset to support reproducibility and model generalization. By offering a comprehensive suite of annotated and augmented images, this dataset provides a valuable resource for developing and refining computer vision models geared toward sunflower detection, maturity evaluation, and yield prediction, ultimately advancing sustainable farming practices and decision-making tools in agricultural research.

Specifications Table

**Table:** 

Subject	Agriculture and biological science
Specific subject area	Computer vision techniques for the detection of sunflowers and its maturity, and yield estimation
Type of data	Image, and XML file
Data collection	We gathered data from various regions in Bangladesh, starting with Nagoriakandi in Narsingdi District on January 16, 2024, and then at Amjhupi vegetables and pulses seed production farm in Meherpur district on February 7, 2024. Our data collection process involved DJI Mavic Mini 2 Drone equipped with a 12MP camera featuring a 1/2.3-inch sensor. Mini 4 K can record up to 4 K video, while the Mini 2 SE can record up to 2.7 K video. Our dataset includes footage of the BARI Surjomukhi-3 variant under various growth conditions (healthy, diseased, mature) and orientations such as facing the sun, facing backwards, and facing downwards. In addition, we included different weather conditions (sunny, foggy) during the data collection process to improve the model's ability to generalise in real-world situations. To ensure accurate labelling for the training process, we utilised Roboflow platform's image annotation tool. Our initial dataset consisted of 1649 sunflower images, which we later augmented using seven different techniques, resulting in a final dataset of 4287 images.
Data source location	Institution: East West University City/Town/Region: Nagoriakandi, Narsingdi, and Amjhupi, Meherpur Country: Bangladesh Latitude and longitude: Nagoriakandi, Narsingdi: Latitude 23.906801° N, Longitude 90.710563° E Amjhupi, Meherpur: Latitude 23.744897° N, Longitude 88.69174° E
Data accessibility	Repository name: Mendeley Data Data identification number: 10.17632/txct4k36ct.1 Direct URL to data: https://data.mendeley.com/datasets/txct4k36ct/1

## 1 Value of the Data


 

• Drone-captured, high-resolution images meticulously annotated for sunflower detection, growth stage, and health conditions enable the development of precise computer vision models [1].
• Unlike conventional drone images taken from overhead perspectives, the dataset includes images captured at lower, eye-sight-level angles. This allows for detailed analysis of individual sunflower plants, including close-up detection of growth stages, leaf conditions, disease symptoms, and plant orientations [2].
• The near-ground perspective complements overhead imagery by providing a more nuanced view of the crop, enabling the development of hybrid systems that integrate both high-level field analysis and close-up plant-level assessment [3].
• By incorporating a variety of angles, including those closer to the plants, the dataset enhances the robustness and adaptability of machine learning models, preparing them for diverse real-world conditions and use cases [4].
• The dataset aids in creating decision-support tools for farmers, facilitating accurate yield predictions, timely interventions, and improved resource management [5,6].
• Its standard Pascal VOC format and comprehensive augmentation techniques make the dataset easily integrable with a wide range of machine learning frameworks, advancing future research in agricultural computer vision [7].

## 2 Background

The creation of this dataset was driven by the need to enhance agricultural practices in Bangladesh through the adoption of advanced technology, specifically targeting the cultivation of sunflowers. This dataset is designed to propel advancements in precision agriculture [4]. Utilizing drone, we captured high-resolution images to develop a comprehensive resource for the detection and assessment of sunflowers across various conditions. The data collection process was thorough, involving the use of a DJI Mavic Mini 2 drone to ensure broad coverage and capture detailed imagery of the sunflower fields. Following this, the images were meticulously annotated on the Roboflow platform, where each sunflower instance was precisely labeled to aid in the training of effective machine learning models. This dataset stands as a crucial resource for research, providing empirical data essential for the development and validation of models aimed at sunflower detection and yield prediction, thereby significantly enhancing both the scope of agricultural research and its practical applications [8].

## 3 Data Description

**Fig. 1:**
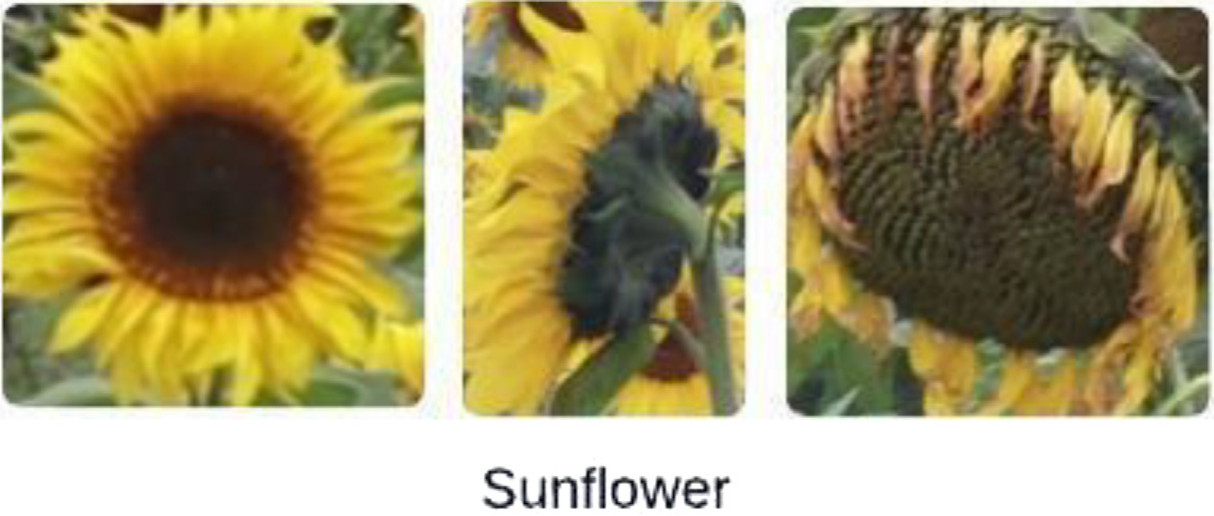
First image shows a sample image of a sunflower. Second image shows a sample of a sunflower's backside. Third image shows a sample image of a matured sunflower.

For this dataset, the total amount of collected data is 1649 annotated sunflower images. The annotations were done using Roboflow [10] which is a web-based data annotation tool. Since the dataset was collected from different angles of the sunflower and from different time periods, the images contained bounding boxes of sunflower class labels. Fig. 1 shows some sample images of this class.

**Fig. 2:**
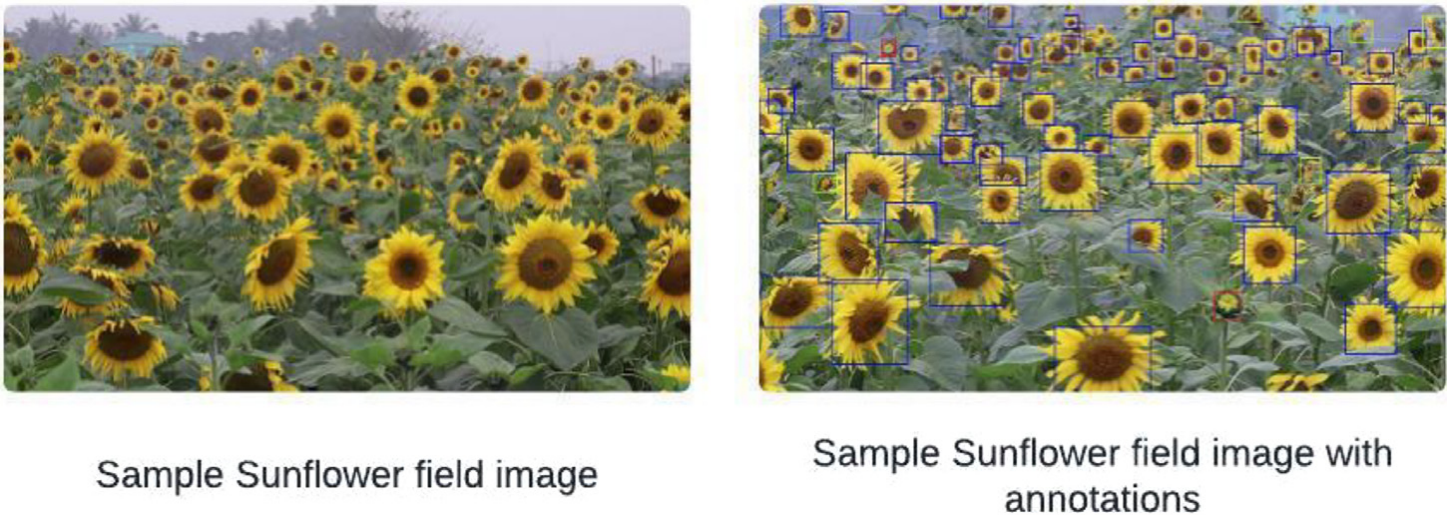
First image shows a sample image of a sunflower field. Second image shows a sample of a sunflower field with annotations.

The images in this dataset are stored in JPG format, and the annotations are provided in Pascal VOC XML format. The Pascal VOC format specifies the bounding boxes of each annotation by the coordinates of the top-left corner along with the width and height, offering a structured way to identify object locations. Each annotation file also contains the image ID and the associated label names, facilitating precise image categorization. Fig.-2 showcases a sample image from the sunflower field, presented both with and without annotations, to illustrate the clarity and utility of this annotation approach.

Since the dataset was collected using different devices all the images needed initial processing to bring them under the same state. After initial pre-processing of the images, augmentations were applied to increase the quantity and variety of the dataset. From different use cases and practical examples, seven types of augmentation were used, such as rotation, Horizontal-flip, hue, exposure, brightness, and saturation. This augmentation gives the dataset more diversity in quality, bringing the dataset to a more realistic state comparable to simulating real-world instances when used to train models. It helps the model to understand the features in different scenarios like the change in lighting in different weather conditions or time, change in perspective and angle of view.

**Fig. 3:**
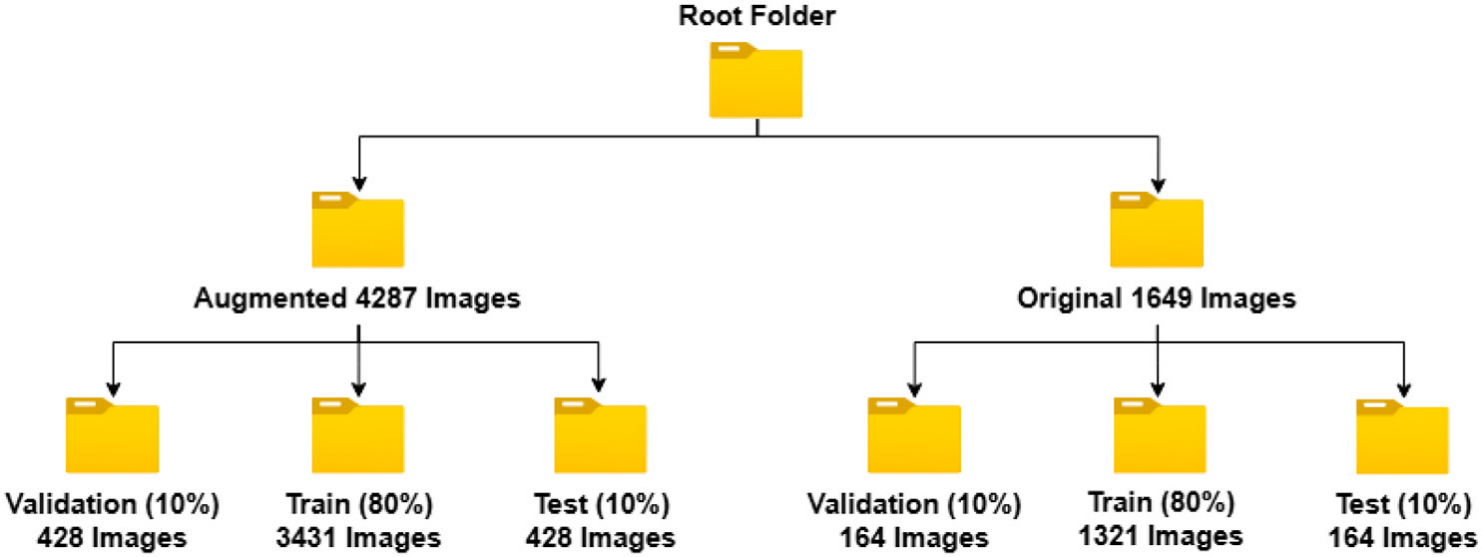
The folder structure of the dataset.

We have separated the augmented and pre-processed dataset in train (80 %), test(10 %) and validation(10 %) subsets. This way the dataset can be directly used for any sort of machine learning or deep learning model training without splitting. In the following Fig. 3 we have shown a basic view of the folder structure used for this dataset. In the root folder, the dataset is divided in 2 folder sections. One contains all the original images, and the other contains the augmented images. All the image folder also contains their Pascal VOC annotations files corresponding to the images. From Fig. 3 we can see that the first folder contains 1649 base image dataset and the second contains a total of 4287 augmented images. In addition to providing the annotations in Pascal VOC format, we have included a link to the Roboflow platform^1^ within the dataset repository on Mendeley Data. This platform allows users to convert the annotations into other formats, including YOLOv5, YOLOv11, and other machine learning frameworks, ensuring broader compatibility and usability.

## 4 Experimental Design, Materials and Methods

**Fig. 4:**
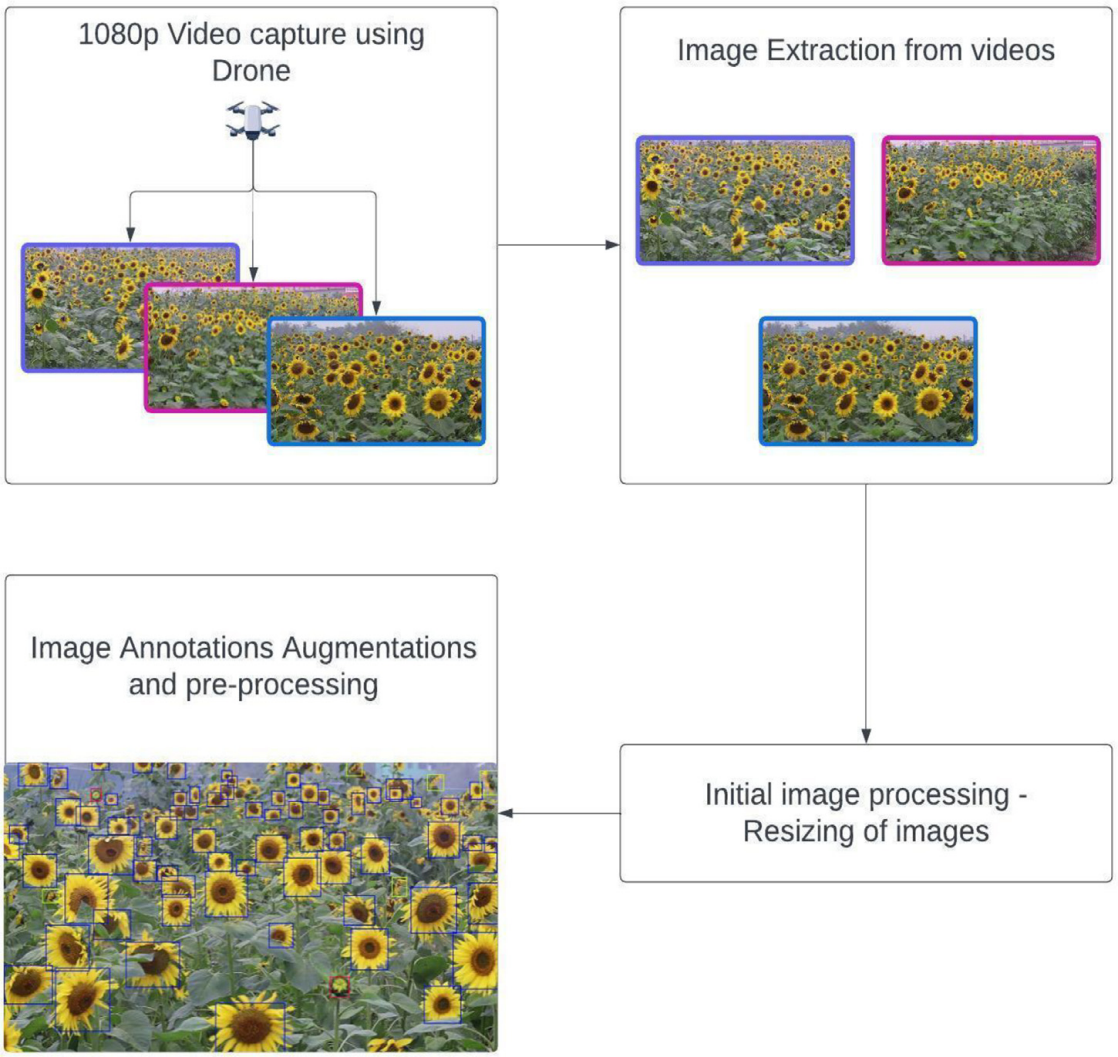
Top level view of the Workflow for the experimental setup.

The experimental setup for data collection and processing involved several key steps. Initially, videos of sunflower fields were captured using a DJI Mavic Mini 2 drone equipped with a 12MP camera featuring a 1/2.3-inch sensor. Mini 4 K can record up to 4 K video, while the Mini 2 SE can record up to 2.7 K video, operating at Full HD resolution (1920×1080 pixels) at 30 frames per second. These videos were taken over the fields of Nagoriakandi, Meherpur, and Amjhupi vegetables and pulses seed production farms. From these videos, images were extracted for further analysis. The next phase involved an initial preprocessing of these images to standardize the resolution, ensuring uniformity across the dataset which is crucial for effective model training. This preprocessing was performed using the Roboflow platform, which also facilitated the annotation of the images. Further image augmentation was then applied to enhance the dataset's diversity and robustness. Fig. 4 provides a comprehensive overview of the entire experimental setup process.

### 4.1 Data collection pipeline

**Fig. 5:**
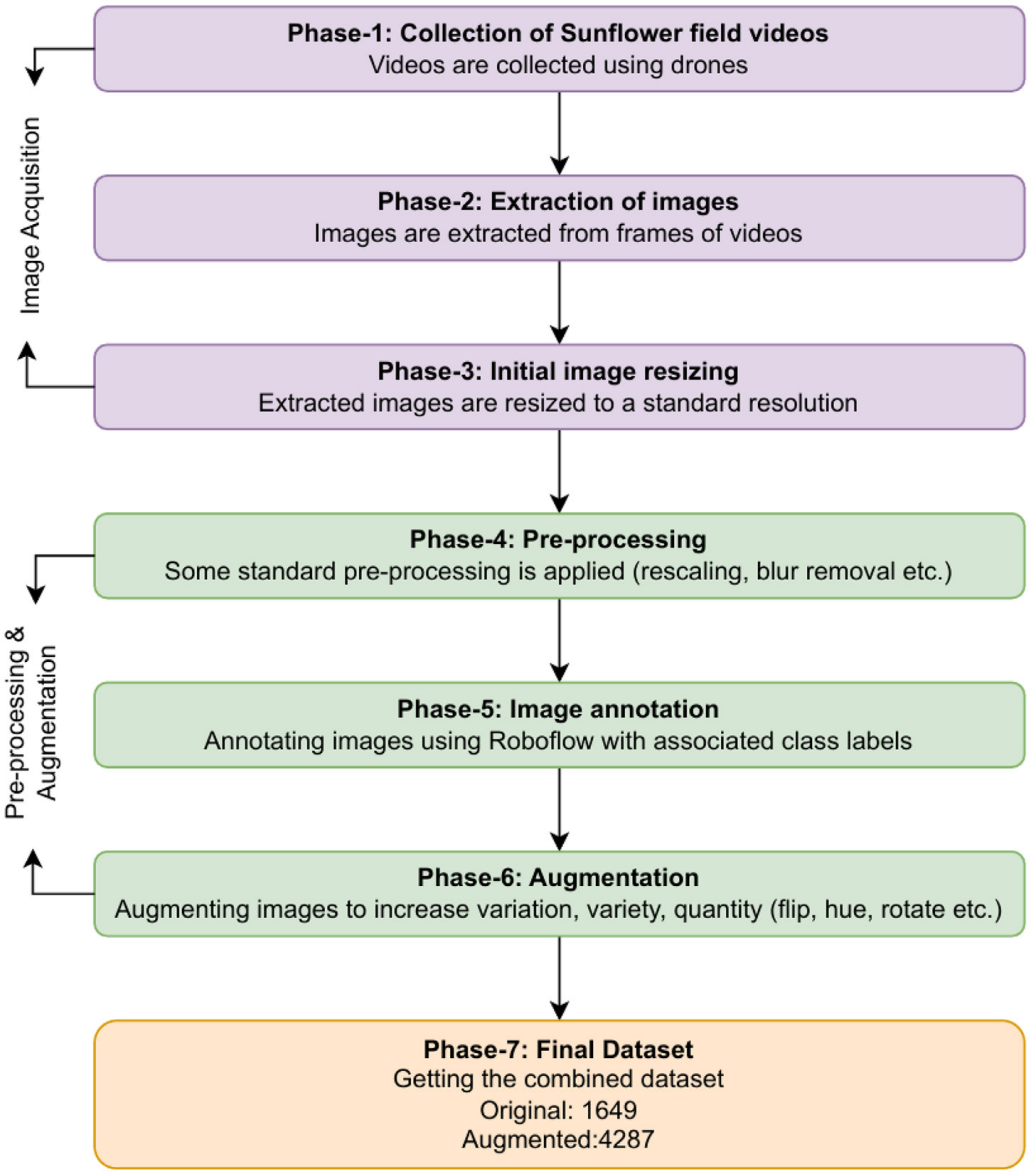
Data collection pipeline.

The data collection goes through a few processes to get to the final stage. The process is divided in two stages, 1st image acquisition and 2nd Pre-processing & Augmentation stage. These stages have further phases. The image acquisition stage is three phases, collection of sunflower field videos, extraction of images and initial resizing of images. In the Pre-processing & Augmentation stage there are, pre-processing phase, annotation phase, augmentation phase and final dataset distribution phase. In Fig. 5 the total pipeline of the process is shown where the pipeline consists of a total of seven phases which are distributed in two stages. The image acquisition stage began by identifying two regions in Bangladesh with sunflower fields: Nagoriakandi, Narsingdi District, and Amjhupi vegetables and pulses seed production farm, located in Amjhupi, Meherpur district. Data capture occurred at these sites on separate dates, January 16th and February 7th, 2023 respectively.

**Fig. 6:**
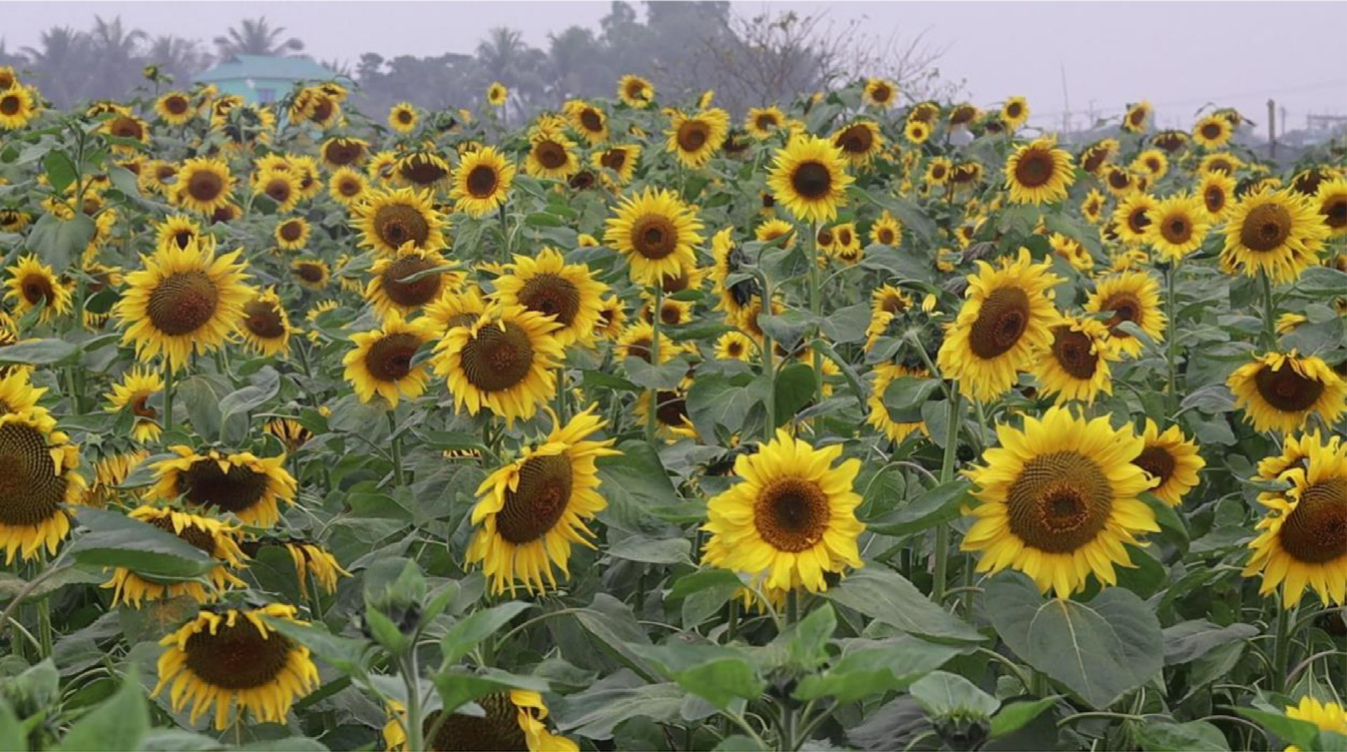
Extracted frame from Drone video.

For image acquisition, a DJI Mavic Mini 2 Drone was employed: This aerial platform provided a wider perspective, allowing for the capture of vast areas of the sunflower fields in a single frame. We have configured the drone at Full HD (1920×1080 pixels) resolution at 30 frames per second to capture the video footage. The primary data collected across both locations consisted of video footage showcasing the sunflower fields. Video was captured from different sunflower fields from three different locations each during different weather and time of day. A total of 58 videos averaging around 2–3 min were captured from these locations. Then images were extracted from video frames (One frame per second). After extraction all the frames go through an initial resizing so that all the images get a standard resolution. Fig. 6 shows Sample Image of extracted frame. (This figure could depict a representative image of a sunflower field captured during data collection).

The next stage is about data processing. Here the extracted images are annotated and cleaned. Any images containing anomalies, or errors are filtered. Misaligned, incorrect or wrong classified Bounding boxes are removed. Since a semi-supervised annotation process is followed, the cleaning process is very crucial. After annotation some preprocessing phases and augmentations are applied for increasing dataset variety, quantity and diversity.

### 4.2 Image preprocessing and annotation

A comprehensive range of preprocessing and augmentation methods were employed to enhance the dataset and ensure it simulates real-world scenarios effectively. Before applying augmentations, the images underwent the following preprocessing steps:

• **Resizing:** All images were resized to 640×640 pixels to standardize input dimensions for model training. This resizing was performed using the “Fit within” method on the Roboflow platform, ensuring that the source image was scaled to fit within the desired dimensions while maintaining the original aspect ratio. Any padding introduced during this process was filled with black pixels.
• **Normalization:** Pixel values were normalized to a range of [0, 1] by dividing each pixel value by 255. This normalization ensures consistent intensity values across the dataset, which facilitates effective model training.
• These techniques are crucial for enriching the dataset's diversity and improving model robustness. The following six key procedures were applied:
• **Saturation Adjustment** [9] **(Between −25 % and +25 %)**: This method alters the image's color intensity within a specified range, enriching the dataset's diversity and enhancing the model's adaptability to varying light conditions.
• **Brightness Modulation** [9] **(between −25 % and +25 %)**: By adjusting the overall lightness or darkness of the images, this technique not only diversifies the dataset but also increases the model's resilience against different light sources.
• **Exposure Variation** [9] **(between −10 % and +10 %)**: This approach modifies how much light the camera captures, which helps in creating a more varied dataset and fortifying the model against fluctuations in lighting.
• **Horizontal Flip** [9]: This simple yet effective method creates a mirror image of the original photo by flipping it horizontally, thereby increasing the dataset's variability.
• **90° Rotation** [9]: Rotating the image by 90°, either clockwise or counterclockwise, adds to the variety of the dataset and offers different perspectives of the same scene.
• **Hue Correction** [9] **(between −20° and +20°)**: Adjusting the hue, or the color tone reflected by the objects, enhances the model's ability to handle variations in environmental lighting conditions.

**Fig. 7:**
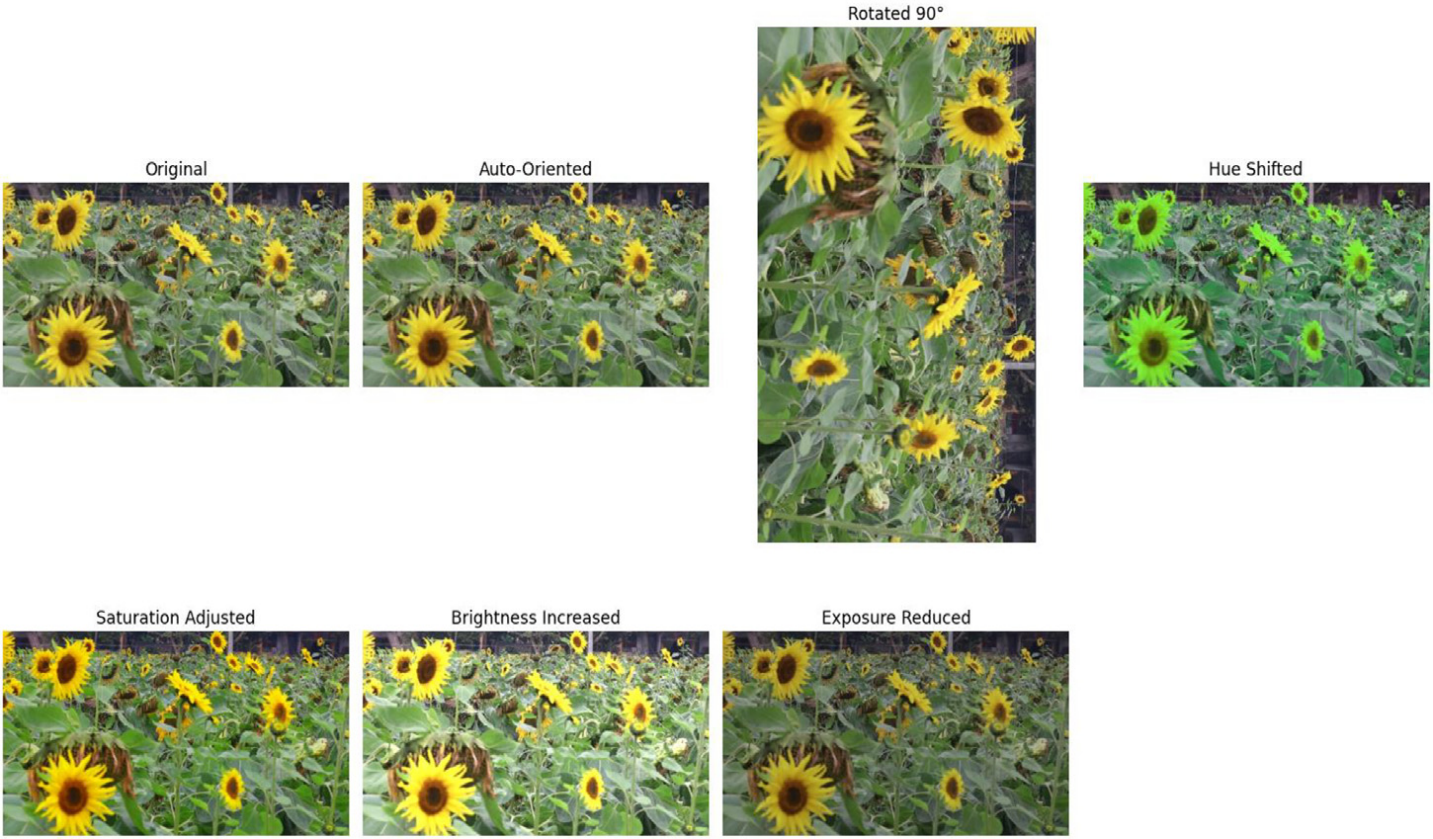
Augmented Image with Multiple Augmentation Applied: This image has undergone auto-orientation and a 90° rotation. Additional adjustments include a hue shift of −18°, a saturation reduction of 22 %, an increase in brightness by 10 %, and a slight reduction in exposure by 1 %.

During the augmentation process, altering images through flipping aids in enhancing the model's adaptability to varied perspectives, such as observing sunflowers from different angles. Additionally, rotating images contributes to the model's resilience to perspective changes. Modifying attributes like hue, saturation, brightness, and exposure can bolster the model's capacity to handle fluctuations in lighting conditions. Fig. 7 illustrates augmented image with multiple transformations. Each chosen image is then resized randomly, fostering diversity in the augmented images and ensuring variability in object sizes. Subsequently, the resized images are combined to form a new image. Adjustments to the bounding boxes of the objects in the original images are made and transferred to the new composite image. Finally, a random crop is taken from the composite image, further enhancing diversity and ensuring varied object placements. Table 1 summarizes the number of images altered by each augmentation technique from the total of 4287 augmented images.

**Table 1:** Summary of augmentation techniques applied to the sunflower dataset, detailing the number of images altered by each technique and their percentage contribution to the total augmented dataset (4287 images). The total number of augmentations exceeds 4287 because multiple augmentations were applied to individual images.

Augmentation technique	Number of images altered	Percentage of total augmented images
Rotation	1072	25 %
Horizontal Flip	1072	25 %
Hue Adjustment	1072	25 %
Saturation Adjustment	1072	25 %
Brightness Adjustment	1072	25 %
Exposure Adjustment	1072	25 %

Here's we have pointed out some data augmentation benefits for our sunflower detection dataset models:

• **Increased Robustness to Viewing Angles:** Techniques like flipping and rotating images make the model perform well even when encountering sunflowers from different angles. Imagine a model trained to identify sunflowers in a field. Flipping and rotating images during training will help it recognize sunflowers regardless of whether they're facing left, right, or at an angle.
• **Improved Lighting Condition Handling:** By adjusting brightness, hue, saturation, and exposure, data augmentation helps models handle variations in lighting. This is crucial because sunflowers experience different lighting conditions throughout the day and across seasons.

## Limitations

Despite its extensive annotations and diverse augmentations, the dataset has some limitations that could affect its generalizability and usability:

• **Geographical Bias:** The dataset was collected from two regions in Bangladesh (Nagoriakandi, Narsingdi, and Amjhupi, Meherpur), which may not represent the diversity of sunflower cultivation across different climates and soil conditions. Future work will involve expanding data collection to include additional locations to enhance geographical diversity.
• **Weather Conditions:** Images were captured under specific weather conditions (sunny and foggy), which do not encompass all possible environmental scenarios, such as overcast or rainy weather. Expanding weather scenarios in future data collection efforts will improve the dataset's robustness.
• **Limited Variability in Crop States:** While the dataset includes sunflowers in various stages (healthy, diseased, mature), it does not include certain stress conditions (e.g., drought, pest infestations). We aim to include such conditions in future iterations to improve model generalization.
• **Background Removal:** The current dataset does not include an additional set of images with backgrounds removed. As future work, we aim to create such a set, focusing solely on sunflower instances to reduce noise and potentially improve object detection accuracy. Background removal will be achieved using semantic segmentation techniques. In the current dataset, users can leverage the Roboflow platform for similar preprocessing tasks, including background removal, to obtain customized outputs for their specific use cases.
• **Additional Metadata:** The dataset lacks certain metadata, such as days since planting and detailed environmental parameters (e.g., temperature, humidity), which could be valuable for researchers. We consider the inclusion of such metadata as part of our future work to provide a richer dataset for advanced analysis.

## CRediT Author Statement

**Md. Shafayat Hossain:** Supervision, Writing - Review & Editing, Conceptualization. **Dr Mohammad Rifat Ahmmad Rashid:** Methodology, Software, Formal Analysis, Data Curation, Validation. **MD Fahim**: Data Curation, Investigation, Writing - Original Draft, Resources. **Tahzib-E-Alindo:** Project administration, Writing - Original Draft, Writing - Review and editing, Data Curation. **Md Sawkat Ali:** Visualization, Data Curation, Writing - Original Draft, Writing - Review & Editing. **Dr. Maheen Islam:** Formal analysis, Validation, Data Curation. **Dr. Mohammad Manzurul Islam:** Data Curation, Writing - Original Draft, Writing - Review & Editing. **Dr. Md. Hasanul Ferdaus:** Supervision, Funding acquisition, Conceptualization. **Nishat Tasnim Niloy:** Supervision, Funding acquisition, Validation.

## Data Availability

Mendeley DataSunflower image dataset from Bangladesh (Original data)

## Footnotes

1 https://universe.roboflow.com/sunflower-detection/final-1yr0d.
